# A Comparison of Wound Healing Rate Following Treatment with Aftamed and Chlorine Dioxide Gels in Streptozotocin-Induced Diabetic Rats

**DOI:** 10.1155/2012/468764

**Published:** 2012-05-15

**Authors:** Fouad Al-Bayaty, Mahmood Ameen Abdulla

**Affiliations:** ^1^Faculty of Dentistry, University Technology Mara, 40450 Shah Alam Selangor, Malaysia; ^2^Faculty of Medicine, University of Malaya, 50603 Kuala Lumpur, Malaysia

## Abstract

*Background and Purpose*. This study aimed to evaluate the wound healing activities of Aftamed and chlorine dioxide gels in streptozotocin-induced diabetic rats. Experimental Approach. Forty-eight Sprague Dawley rats were chosen for this study, divided into 4 groups. Diabetes was induced. Two-centimeter-diameter full-thickness skin excision wounds were created. Animals were topically treated twice daily. Groups 1, the diabetic control group, were treated with 0.2 mL of sterile distilled water. Group 2 served as a reference standard were treated with 0.2 mL of Intrasite gel. Groups 3 and 4 were treated with 0.2 mL of Aftamed and 0.2 mL of chlorine dioxide gels respectively. Granulation tissue was excised on the 10th day and processed for histological and biochemical analysis. The glutathione peroxidase ,superoxide dismutase activities and the malondialdehyde (MDA) levels were determined. Results. Aftamed-treated wounds exhibited significant increases in hydroxyproline, cellular proliferation, the number of blood vessels, and the level of collagen synthesis. Aftamed induced an increase in the free radical-scavenging enzyme activity and significantly reduced the lipid peroxidation levels in the wounds as measured by the reduction in the MDA level. Conclusions. This study showed that Aftamed gel is able to significantly accelerate the process of wound healing in diabetic rats.

## 1. Introduction

Wound healing is a dynamic process involving cellular, molecular, biochemical, and physiological phenomena that result in connective tissue repair and fibrous scar formation and lead to the restoration of the anatomical continuity and functional status of the skin [1]. The wound-healing process consists of four highly integrated and overlapping phases: hemostasis, inflammation, proliferation, and tissue remodeling or resolution [2]. These phases and their biophysiological functions must occur in the proper sequence, at a specific time and must continue for a specific duration at an optimal intensity [3]. There are many factors that can affect wound healing by interfering with one or more phases in this process, thus causing improper or impaired tissue repair.

In individuals with diabetes mellitus, the rate of wound repair is slow [4]. The underlying mechanisms of defective wound repair in diabetic patients are not completely understood, but it is thought that all phases of the healing process are disrupted. Indeed, delayed collagen synthesis, impaired epithelialization, and reduced angiogenesis have been observed during the proliferative phase of the healing process [5]. Others have reported that fibroblasts do not produce adequate amounts of extracellular matrix and that keratinocytes do not reepithelialize the wound [6]. As a result, the skin microvasculature becomes damaged, tissue ischemia ensues, and chronic diabetic wounds develop [7]. Increased apoptosis is a common phenomenon in chronic diabetic wounds [8]. Farahani and Kloth [9] showed that the rate of wound closure is associated with the extent of apoptosis in the wound: an increased rate of wound closure is associated with a reduced extent of apoptosis. Angiogenesis increases the delivery of oxygen and other nutrients that are necessary for local collagen synthesis [10]. Poor wound healing associated with diabetes mellitus is characterized by decreased wound collagen content and diminished wound tensile strength [11]. Diabetic wounds result in significant morbidity, prolonged hospitalization, and enormous health-care expenses [12]. Better ways to treat diabetic wounds are needed to develop new therapeutic strategies. Hyaluronic acid is a naturally occurring physiological constituent of connective tissue, especially in the gingival mucosa. Hyaluronic acid is a glycosaminoglycan composed of repeating disaccharides of D-glucuronic acid and N-acetylglucosamine [13]. Hyaluronidase is able to break down the proteoglycans in the ground substance of connective tissues and invade periodontal structures. High-molecular-weight hyaluronic acid can therefore exert an efficient antihyaluronidase effect as a result of its physiological macroaggregating activity [14]. Hyaluronic acid also plays an important role in tissue repair and exerts a protective effect. Under normal conditions, the proteoglycans in the ground substance of gingival mucosa connective tissue represents an effective barrier against bacterial invasion and against the spread of bacterial toxins. If this barrier is damaged, the topical application of hyaluronic acid can help reconstruct the barrier due to the macro-aggregating effect of hyaluronic acid on proteoglycans [14]. The macro-aggregating effect of hyaluronic acid on proteoglycans inhibits the development of edema, an effect that is also known as the antiedematous effect [14]. Aftamed gel, which is composed of high-molecular-weight hyaluronic acid (240 mg/100 g), is an exciting new product that is very effective and accelerates tissue healing, in addition to possessing anti-inflammatory and antiedematous characteristics [15]. Gels containing hyaluronic acid have a number of significant applications in dentistry, for example, in the repair, healing, and regeneration of gum tissue as an integral element of the treatment of gingivitis and periodontitis [16]. A common use of chlorine dioxide in drinking water has been the control of tastes and odors associated with algae and decaying vegetation. Chlorine dioxide is also effective in destroying taste- and odor-producing phenolic compounds. Chlorine dioxide aids in relieving pain after wisdom tooth removal and enhances the healing process following oral surgical procedures [17]. According to a study performed by Eddy et al. [18], chlorine dioxide is capable of completely killing *Enterobacter faecalis* within 30 minutes at higher concentrations, making chlorine dioxide an effective endodontic irrigant. Thus, given the fact that chlorine dioxide has an antibacterial effect, several companies have used chlorine dioxide as the main ingredient in oral medicines, especially medicines for the treatment of periodontal disease. However, the efficacy of chlorine dioxide in reducing and preventing dental plaque formation has not previously been extensively studied. A new product, Penetrater chlorine dioxide oral gel offers a new procedure for the treatment of inflamed periodontal pockets and gingivitis. There have been no reports published regarding the comparison of the wound-healing effects of Aftamed gel (high-molecular-weight hyaluronic acid 240 mg/100 g) and those of chlorine dioxide gel. This lack of published data encourages the assessment of the rate of wound healing in response to treatment with hyaluronic acid gel and chlorine dioxide gel and the mechanisms by which these gels promote healing in rats.

## 2. Materials and Methods

### 2.1. Intrasite Gel

Intrasite gel was purchased from the University Malaya Medical Center Pharmacy and was used as a positive control. Intrasite gel is a colorless transparent aqueous gel that contains a modified carboxymethylcellulose polymer (2.3%) and propylene glycol (20%), which has bacteriostatic properties. In addition, propylene glycol acts as a humectant and a preservative, thus preventing the gel from drying out and improving handling. Intrasite gel is an amorphous hydrogel that gently rehydrates necrotic tissue and facilitates autolytic debridement while loosening and absorbing slough and exudates, allowing effective wound healing. Intrasite gel is also designed for wounds that are granulating and epithelizing. This gel can also be used to maintain the optimum moisture level of the wound environment during the later stages of wound closure. Intrasite gel is nonadherent and does not harm viable tissue or the skin surrounding the wound, thus making it ideal for every stage in the wound management process. In this study, 0.2 mL of Intrasite gel was applied topically twice daily to the wounds of Group 2 rats (Intrasite gel is a trademark of Smith and Nephew Ltd.) [19].

### 2.2. Aftamed (Hyaluronic Acid Gel)

Hyaluronic acid is characterized by a high viscosity. Hyaluronic acid has a large number of essential functions and is found naturally in a number of body tissues. Aftamed also contains polycarbophil, a protein that attaches to wet mucosa. This attachment provides direct mechanical protection and keeps the hyaluronic acid in place on the wound. Polycarbophil is harmless. Hyaluronic acid plays an integral role in maintaining and regulating the moisture level in tissues and facilitates the transport of nutrients into cells and the removal of metabolic waste. Hyaluronic acid is a glycosaminoglycan with anti-inflammatory and antiedematous properties. Aftamed high-molecular-weight hyaluronic acid 240 mg/100 g gel was purchased from the dental suppliers of Pharmaniaga Manufacturing Bhd Kuala Lumpur, Malaysia.

### 2.3. Chlorine Dioxide Oral Gel

Penetrater chlorine dioxide oral analgesic gel was purchased from Frontier Pharmaceutical, Inc. Melville, NY, USA.

### 2.4. Lignocaine HCl (2%, 100 mg/5 mL)

Lignocaine is a local anesthetic and was purchased from the Experimental Animal House, Faculty of Medicine, University Malaya (delta Veterinary Laboratory PTY LTD, NSW 20011). One milliliter of lignocaine was injected subcutaneously.

### 2.5. Streptozotocin

Streptozotocin, a pancreatic beta-cell toxin, was purchased from Sigma (St. Louis, MO). This drug was dissolved and injected intraperitoneally at a dose of 50 mg/kg [20].

### 2.6. Experimental Animals

Healthy adult male *Sprague Dawley* rats were obtained from the experimental animal house, Faculty of Medicine, University of Malaya. The rats were divided randomly into 4 groups of 12 rats each. Each rat that weighed between 220 and 250 g and was housed separately (one rat per cage). The animals were maintained on a standard pellet diet and tap water. The study was approved by the Research Committee on the Ethical Use of Animals in Research (UiTM Care) Universiti Teknologi MARA Ethic no. 600-FF(PT,5/2) 20/7/2009. All animals received care according to the criteria outlined in the “Guide for the Care and Use of Laboratory Animals” prepared by the National Academy of Sciences and published by the National Institutes of Health.

### 2.7. Diabetic Animals

After overnight fasting, diabetes mellitus was induced in all groups by a single injection of streptozotocin (STZ; 50 mg/kg, i.p.) prepared in citrate buffer (0.1 M, pH 4.5) [20]. Blood was drawn from the tail on the 7th day after the STZ injection, and the blood glucose levels were estimated using a glucometer (Ames, Bayer Diagnostic). The blood glucose levels were used to confirm the development of diabetes. Rats with elevated blood glucose levels (≥150 mg/dL) were considered diabetic and were subject to the wounding procedure. The blood glucose levels were estimated at the time of the creation of the wounds.

### 2.8. Experimentally Induced Excision Wounds in Diabetic Rats

Wounds were created on the 7th day after the induction of diabetes in all rats. The animals were anesthetized with ketamine (5 mg/kg i.p.) and xylazine (2 mg/kg i.p.). The skin was shaved using an electrical clipper and disinfected with 70% alcohol, and 1 mL of lignocaine HCl was injected. A uniform wound of 2.00 cm in diameter was excised from the nape of the dorsal neck of each rat with the aid of a round seal as described in [21]. Care was taken to avoid injuring the muscle layer, and the tension of skin was kept constant during the procedure. The wound area was measured immediately by placing a sheet of transparent tracing paper over the wound and tracing. The tracing paper was placed on sheet of 1 mm^2^ graph paper, and the wound tracing was traced. The squares were counted, and the area was recorded as described in [22].

### 2.9. Topical Application

The wounds of Group 1 rats were dressed with 0.2 mL of sterile distilled water twice daily; thee rats served as the diabetic control group [23]. The wounds of Group 2 rats were dressed topically twice daily with 0.2 mL of Intrasite gel as a reference. In addition, 0.2 mL of Aftamed gel and 0.2 mL of chlorine dioxide oral were applied topically twice daily to the wounds of rats in Groups 3 and 4, respectively. All animals were sacrificed on day 10 after surgery. The wound closure area of each animal was assessed by tracing the wound on days 1, 5, and 10 after wounding surgery using transparent paper and a permanent marker under light ketamine and xylazine anesthesia as described by Nayak and Pinto-Pereira [24]. The wound areas recorded were measured using graph paper. The percent wound healing values on these days were determined.

### 2.10. Histological Evaluation of Wound Tissues

On day 10 after surgery, skin specimens from the wound areas were fixed in 10% buffered formalin and were processed by a tissue processing machine. The wound area was assessed by staining a 5 *μ*m section with hematoxylin and eosin.

### 2.11. Antioxidant Measurement from Granulation Tissue

Wound tissue was collected on day 10 for measurement of the activities of the antioxidant enzymes glutathione peroxidase (GPx) and superoxide dismutase (SOD). The level of lipid peroxidation was determined by measuring the level of malondialdehyde (MDA) in the granulation tissue from wound tissue area on day 10 after wounding.

### 2.12. Protein Determination

The total protein content of the wound tissue from each animal was determined according to the method of [25]. Briefly, wound tissues on day 10 after wounding were homogenized in 1.15% calcium chloride at a ratio of 1 : 5 (w/v). A 0.1 mL quantity of the homogenate was added to 5 mL of Bradford reagent and mixed, and the absorbance was read at 595 nm against a blank of 0.1 M phosphate buffer, pH 7.4. Standards were treated similarly using BSA at concentrations of 0, 20, 40, 60, 80, and 100 *μ*g/mL in 0.1 M phosphate buffer at pH 7.4.

### 2.13. Glutathione Peroxidase Activity

For the determination of GPx activity in the wound tissue, samples of day-10 wound tissues were prepared by homogenizing these tissues in 1.15% potassium chloride at a ratio of 1 : 5 (w/v). The homogenized samples were then centrifuged at 8,000 rpm for 20 min at 4°C. The resultant supernatant was again centrifuged at 35,000 rpm for 1 h at 4°C.

The level of glutathione peroxidase (GPx) activity was determined using the method of Lawrence and Burk [26]. The reaction mixture contained 2 mL of phosphate buffer (50 mM; pH 7.0), 0.2 mL of EDTA, 0.3 mL of sodium azide (1 mM), 0.1 mL of GSH (1 mM), and 0.1 mL of NADPH (0.2 mM). To this mixture, 0.2 mL of enzyme solution was added. The mixture was then incubated for 5 min at room temperature, and the reaction was started by adding 0.2 mL of 0.25 mM H_2_O. The optical density was measured at 340 nm at 20 min intervals. The enzymatic activity was measured as the micromoles of NADPH oxidized per minute per milligram protein.

### 2.14. Superoxide Dismutase Activity

Homogenized wound tissue was prepared on day 10 after wounding as described for GPx and was analyzed for SOD activity. The SOD activity was determined according to the method of Beyer and Fridovich [27]. The hemolysate was mixed with a substrate mixture consisting of 50 mM phosphate buffer containing 0.1 mM EDTA, pH 7.8, prepared fresh on the day of analysis; L-methionine (0.03 g/mL); nitroblue tetrazolium chloride (NBT*·*2HCl) (1.41 mg/mL); 1% Triton X-100 (vol/vol). The reaction was started by adding 10 *μ*L of riboflavin (4.4 mg/100 mL). The mixtures were mixed and then left in an aluminum box illuminated with two 20-W fluorescent lamps for 7 min, after which time the absorbance was read at 560 nm. The whole procedure was carried out in the dark because both riboflavin and NBT are sensitive to light.

### 2.15. Malondialdehyde Levels

The MDA levels in the day-10 wound tissue homogenate were also measured using TBAR methods to determine the level of lipid peroxidation [28]. Briefly, the tissues were homogenized in 1.15% potassium chloride and diluted 1 : 5 with distilled water before analysis. A 2.5-mL quantity of trichloroacetic acid solution was then added to the hemolysate, which was incubated at room temperature for 15 min. Thiobarbituric acid (1.5 mL) was then added to the mixture, which was then mixed and incubated in a water bath for 30 min. The tubes were then cooled and shaken vigorously for 30 min before adding 4 mL of n-butanol solution. The tubes were then centrifuged at room temperature. The absorbance of the resulting upper layer was then read using a spectrofluorometer (Shimadzu, Kyoto, Japan).

### 2.16. Statistical Analysis

All values are reported as the mean ± S.E.M., and the statistical significance of differences among groups was assessed using one-way ANOVA. A value of *P* < 0.05 was considered significant.

## 3. Results

### 3.1. Wound-Healing Activity in Diabetic Rats

#### 3.1.1. Wound Closure

**Figure 1:**
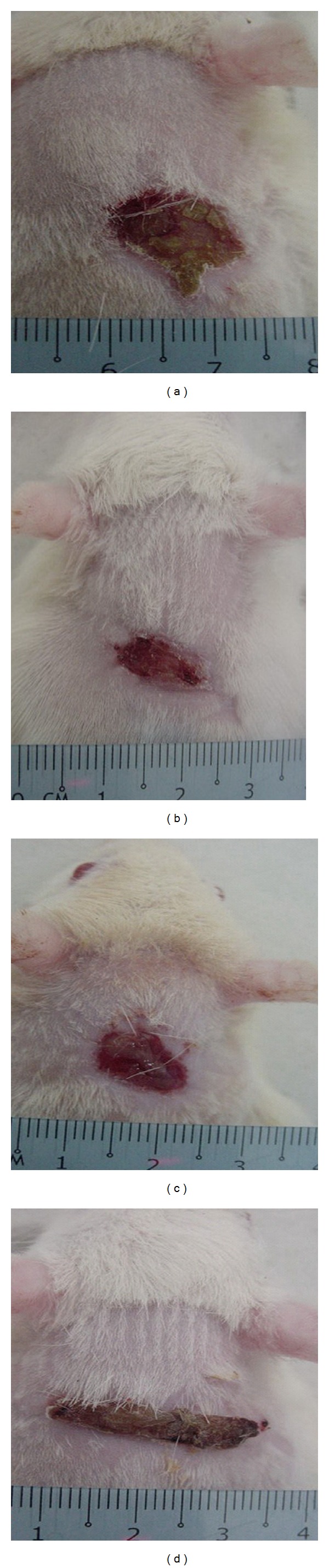
Macroscopic appearance of the wounds on day 10 after surgery in diabetic rats. By visual inspection: (a) The topical application of 0.2 mL sterile distilled water resulted in a wide wound closure area. (b) The topical application of 0.2 mL Intrasite resulted in a smaller wound closure area than that of the control diabetic group. (c) The topical application of 0.2 mL chlorine dioxide resulted in a significantly smaller wound closure area than that of the control diabetic group. (d) The topical application of 0.2 mL Aftamed gel resulted in a significantly smaller wound closure area than that of the control diabetic group.

**Figure 2:**
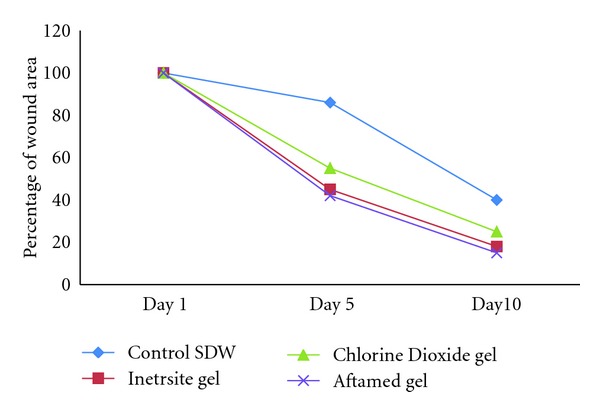
Mean ± SEM of percent wound areas during healing of excision dermal wounds of control, Intrasite gel, choline dioxide gel, and Aftamed gel.

The rate of wound healing was evaluated by an observer blinded to the experimental protocol. Gel treatment resulted in smaller wounds on day 10 after wounding compared with the control treatment in diabetic rats (Figure 1). Gel-treated rats had significantly smaller wound areas on day 10 of healing than the diabetic control rats did, and the wound areas of the rats treated with the Aftamed gel were significantly smaller than those of the chlorine dioxide-treated rats (Figure 1). Throughout the experiment, the percent healing in the diabetic control group was significantly lower than those in the Intrasite gel-treated, Aftamed gel-treated, and chlorine dioxide gel-treated groups (Figure 2). The treatment of the wounds of diabetic rats with gels increased the rate of wound contraction relative to the control treatment, and the wounds in the gel-treated groups were clean and contained bright red healthy granulation tissue (Figure 1). In contrast, the wounds of the diabetic control rats were generally unclean (Figure 1). Visual inspection revealed that the Aftamed-treated wounds appeared to show improved healing and were smaller in size as early as day 5 relative to the wounds in the diabetic control group (Figure 2). However, the percent healing values in Intrasite gel and Aftamed groups were significantly greater than those in the chlorine dioxide gel group (Figure 2).

### 3.2. Histology of Wound Tissues

**Figure 3:**
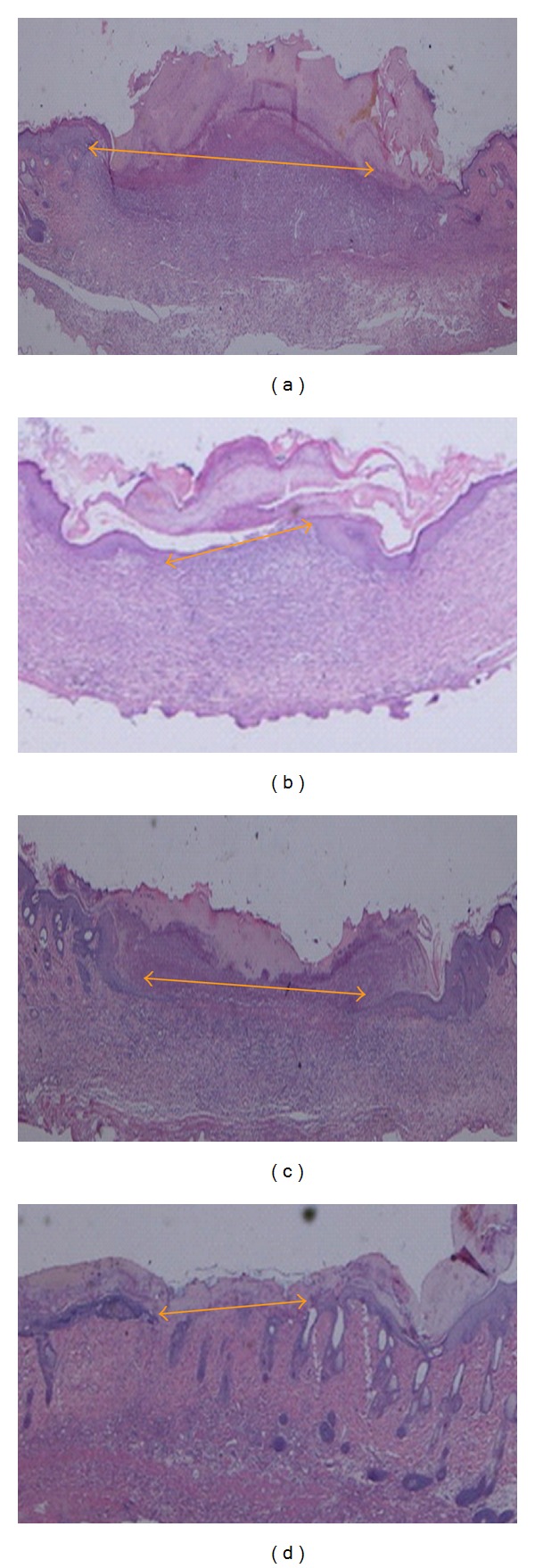
Histological section of the wound tissue on day 10 after surgery in diabetic rats. (a) The topical application of 0.2 mL of sterile distilled water resulted in a wide wound area (arrow). (b) The topical application of 0.2 mL of Intrasite gel resulted in a comparatively smaller wound enclosure than the control treatment did (arrow). (c) The topical application of 0.2 mL of chlorine dioxide gel resulted in a comparatively moderately sized wound enclosure relative to that of the control treatment (arrow). (d) The topical application of 0.2 mL of Aftamed gel resulted in a comparatively smaller wound enclosure than the control treatment did (arrow) (H & E stain 2×).

**Figure 4:**
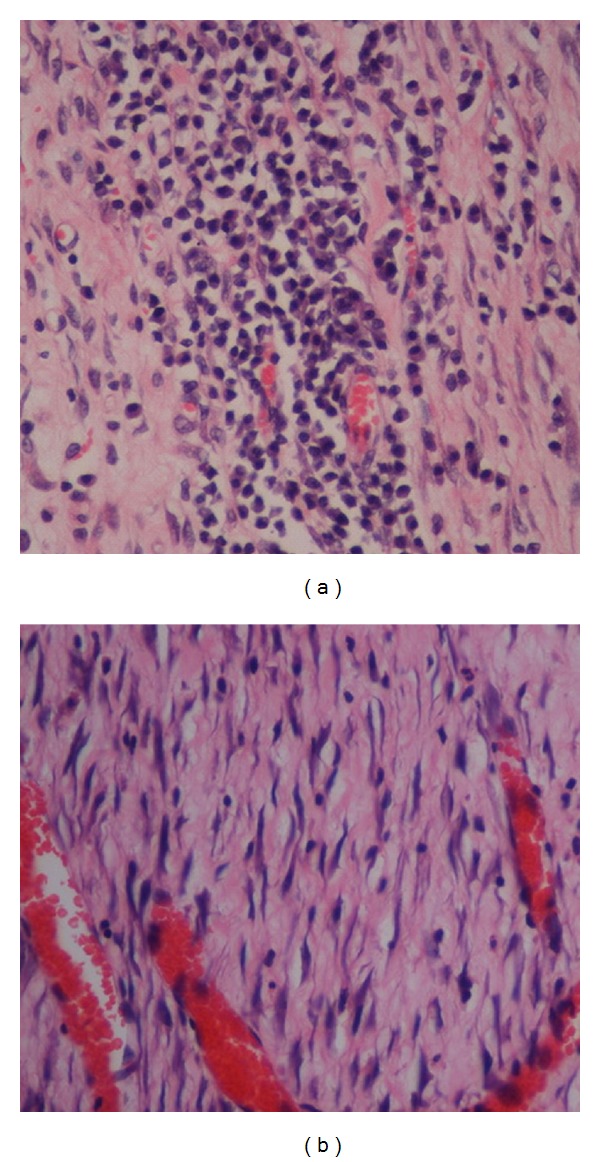
High-magnification images of the histological sections of wound enclosure on day 10 after surgery in diabetic rats that are shown in Figures 3(a) and 3(d). The tissue shown in (a) harbors comparatively more inflammatory cells (mononuclear cells) than the tissue shown in (d). The tissue shown in (d) shows comparatively more collagen deposition in the wound area and increased angiogenesis relative to the tissue shown in (a) (H & E stain, magnification 40×).

The histology of the wound tissues on day 10 after wounding was evaluated by an observer blinded to the experimental protocol. In gel-treated groups, the wound areas were smaller than that of the diabetic control group (Figure 3), and the granulation tissues from the gel-treated groups contained comparatively fewer inflammatory cells and more collagen and more proliferating blood capillaries than the granulation tissues of the diabetic control group (Figures 3 and 4). Gel treatment stimulated and enhanced the deposition of collagen fibers and the formation of new blood vessels in the granulation tissue than the control treatment (Figure 4).

### 3.3. Total Protein Content

**Figure 5:**
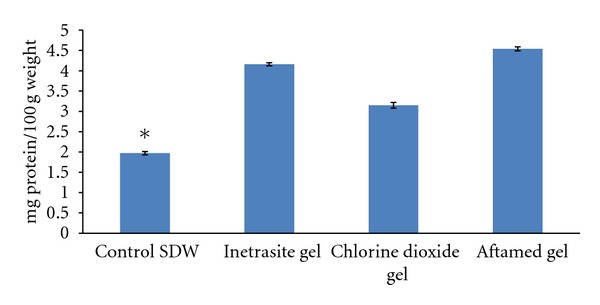
Mean ± SEM of total protein of wound area in day 10- after surgery.

The total protein content of the wound area was significantly increased in the gel-treated groups relative to that in the diabetic control group (Figure 5). Higher protein levels were observed for the diabetic rats treated with Aftamed gel (Figure 5).

### 3.4. Antioxidant Enzyme Activities and MDA Levels in Wound Tissues

The GPx and SOD activities and MDA levels were calculated, taking into account the amounts of protein present in the healing wounds, because these values are indicative of the number of living cells present. The results showed that the activity levels of both enzymes were significantly elevated in rats treated with gels compared with the levels in the diabetic control rats (Table 1). Higher levels were observed in the diabetic rats treated with Aftamed gel. There was a significant reduction in the MDA level observed in the tissue taken from rats treated with Aftamed gel (Table 1).

## 4. Discussion

The primary finding of this study is that the topical application of Aftamed to skin excision wounds in diabetic rats increases the rate of wound repair. Histological analysis of the wound area confirmed visually apparent increases in wound repair, showing that an increase in angiogenesis and increased collagen deposition are the mechanisms underlying the action of topical Aftamed. In addition, gel treatment resulted in a significantly smaller wound area in diabetic rats on day 10 after wounding relative to the control treatment. The amounts of protein in the wound tissue taken from the rats treated with gels were significantly greater than the amounts for the diabetic control rats. The amounts of protein in wounds treated with Aftamed gel or Intrasite gel were significantly increased compared with the amount in the chlorine dioxide gel-treated group. Collagen is a major determinant of the increase in tensile strength of healing wounds [29]. Topically administered Aftamed exerts its beneficial effects on wound healing by stimulating the deposition of collagen and angiogenesis. Angiogenesis increases the delivery of oxygen and other nutrients that are necessary for local collagen synthesis [10]. Wound-healing deficits associated with diabetes are diverse, multifactorial, complex, and interrelated [30] and are believed to be caused by impaired blood flow and impaired oxygen release due to increased blood sugar, decreased collagen, and fibronectin synthesis due to protein malnutrition, impaired local immune, and cell defenses and decreased anabolic activity with decreased levels of insulin and growth hormone. The influx of macrophages and the proliferating mesenchymal cells and capillaries that make up the granulation tissue may also provide substrates for and express inducers of reepithelialization [31]. It has been postulated that applying agents that induce fibroblast and/or endothelial cell proliferation to healing-impaired wounds might increase the rate and degree of granulation tissue formation and stimulate wound repair [32]. In response to tissue loss, fibroblasts proliferate and migrate into the defect until the wound is populated by fibroblasts and extracellular matrix [33]. Similarly, enhanced healing activity has been attributed to increased collagen deposition and angiogenesis [34]. Collagen plays a central role in the healing of wounds, and it is a principal component of connective tissue and provides a structural framework for the regenerating tissue. The stimulation of epithelial cell proliferation and angiogenesis is important for wound healing [35]. Wound contraction occurs early, with the most dramatic wound contraction occurring during days 5–10 after wounding and appears to be dependent on both the number of fibroblasts and the collagen content. Cellular contraction is more important early on than collagen deposition in reducing the diameter of the wound [36]. The delayed healing in the Penetrater chlorine dioxide oral gel group may be due to a lower amount of collagen in the wound tissue and to the lower number of myofibroblasts, which play a crucial role in wound contraction [36].

In the present study, tissue homogenate from diabetic wounds treated with gel showed significant antioxidant activity by decreasing the levels of MDA and by elevating the levels of GPx SOD in response to oxidative stress due to diabetes. SOD converts superoxide into hydrogen peroxide (H_2_O_2_), which is then transformed into water by catalase in lysosomes or by glutathione peroxidase (GPx) in mitochondria [37]. Reduced activities of SOD and GPx were observed in the tissue homogenate of diabetic control rats. GPx is thought to be an important factor in cellular function and the defense against oxidative stress. It was found that dietary GPx suppresses oxidative stress *in vivo*, preventing diabetes-related complications [38]. Lipid peroxidation is found to be an important pathophysiological event in a variety of diseases including diabetes [39]. It is well known that MDA from lipid peroxidation reacts with DNA bases and induces mutagenic lesions [40].

## 5. Conclusion

These results suggest that Aftamed gel has a beneficial effect and plays a major role in diabetic wound healing. The topical application of this gel accelerates wound healing in diabetic rats, and this accelerated healing could be due to increases in the activities of antioxidants (SOD and GPx) in the wound tissue and to a decrease in the MAD level relative to diabetic controls. Our documented findings suggest that Aftamed gel should be used for the treatment and management of diabetic wounds. Such findings encourage further investigation to obtain a greater understanding of the wound healing activity of Aftamed gel in diabetic rats.

## Figures and Tables

**Table 1:** Mean ± SEM of the glutathione peroxidase (GPx) and superoxide dismutase (SOD) activities and the malondialdehyde (MDA) levels in the tissue homogenates of day-10 dermal wounds of the control, Intrasite gel-treated, chlorine dioxide gel-treated, and Aftamed gel-treated diabetic rats.

	No. of animals	Control	Intrasite gel	Chlorine dioxide gel	Aftamed gel
GPx (*μ*/g protein)	6	12.54 ± 0.25^a^	35.17 ± 0.58	24.38 ± 0.32^a^	31.83 ± 1.68^a^
SOD (u/mg protein)	6	19.41 ± 0.42^b^	51.23 ± 0.35^b^	32.67 ± 0.49^b^	49.73 ± 0.44^b^
MDA (nmol/g protein	6	92.94 ± 0.27^c^	13.00 ± 0.26^c^	22.50 ± 0.48^c^	14.50 ± 0.48^c^

All values are expressed as the mean + SEM. Means labeled with different superscripts were significantly different. These differences were significant at the 0.05 level.
