# Corrigendum to “A Comparison of Wound Healing Rate Following Treatment with Aftamed and Chlorine Dioxide Gels in Streptozotocin-Induced Diabetic Rats”

**DOI:** 10.1155/2019/4265081

**Published:** 2019-01-10

**Authors:** Fouad Al-Bayaty, Mahmood Ameen Abdulla

**Affiliations:** ^1^Faculty of Dentistry, University Technology Mara, 40450 Shah Alam Selangor, Malaysia; ^2^Faculty of Medicine, University of Malaya, 50603 Kuala Lumpur, Malaysia

The article titled “A Comparison of Wound Healing Rate Following Treatment with Aftamed and Chlorine Dioxide Gels in Streptozotocin-Induced Diabetic Rats” [1] was found to contain figures from an earlier article by the same authors, N. Al-Henhena et al. [2], where Figure 1(c) in [1] is the same as Figure 1(a) in [2].

An institutional investigation by the University of Malaya found there was no system to index and file data and images to avoid mislabeling and mishandling, which led to errors and duplication of research data. The authors did not thoroughly check the manuscript before submission.

The authors said this was a mistake by their research assistant, with no intent to mislead or fabricate data at any step of the experimental design, execution, and manuscript preparation, and what happened was due to unconscious human error during image selection from the experimental folder of Prof. Abdulla's departmental computer.

The Editorial Board recommended publishing a corrigendum. Corrected Figure 1 is shown below.

## Figures and Tables

**Figure 1:**
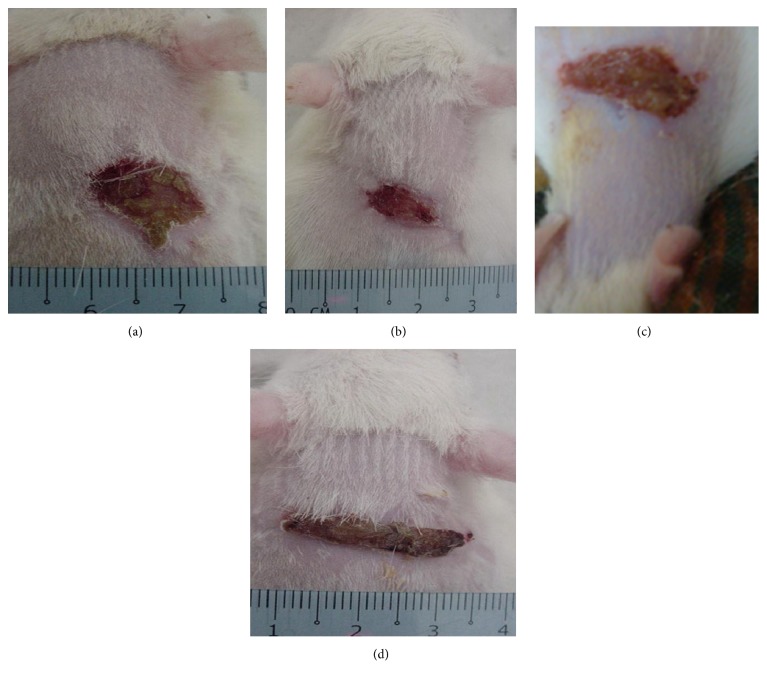
Macroscopic appearance of the wounds on day 10 after surgery in diabetic rats. By visual inspection: (a) The topical application of 0.2 mL sterile distilled water resulted in a wide wound closure area. (b) The topical application of 0.2 mL Intrasite resulted in a smaller wound closure area than that of the control diabetic group. (c) The topical application of 0.2 mL chlorine dioxide resulted in a significantly smaller wound closure area than that of the control diabetic group. (d) The topical application of 0.2 mL Aftamed gel resulted in a significantly smaller wound closure area than that of the control diabetic group.
